# Reverse causal relationship between periodontitis and shortened telomere length: Bidirectional two-sample Mendelian random analysis

**DOI:** 10.3389/fimmu.2022.1057602

**Published:** 2022-12-19

**Authors:** Jiaxin Hu, Jukun Song, Zhu Chen, Jing Yang, Qianhui Shi, Fuqian Jin, Qiyuan Pang, Xingtao Chang, Yuan Tian, Yi Luo, Liming Chen

**Affiliations:** ^1^ Guiyang Hospital of Stomatology, Stomatology Hospital of Guizhou University, Guiyang, Guizhou, China; ^2^ School of Stomatology, Zunyi Medical University, Zunyi, Guizhou, China; ^3^ The Affiliated Stomatological Hospital & Stomatology of Guizhou Medical University, Guizhou Medical University, Guiyang, China; ^4^ Guizhou Provincial People's Hospital, Guiyang, Guizhou, China

**Keywords:** telomere length, periodontal disease, Mendelian randomization, causality, genome-wide association studies, biological aging, inflammation

## Abstract

**Background:**

Observational studies have demonstrated a link between shortened telomere lengths(TL) and chronic periodontitis. However, whether the shortened TL is the cause or the result of periodontitis is unknown.Therefore, our objective was to investigate a bidirectional causal relationship between periodontitis and TL using a two-sample Mendel randomized (MR) study.

**Methods:**

A two-sample bidirectional MR analysis using publicly available genome-wide association study (GWAS) data was used. As the primary analysis, inverse variance weighting (IVW) was employed. To identify pleiotropy, we used leave-one-out analysis, MR-Egger, Weighted median, Simple mode, Weighted mode, and MR pleiotropy residual sum and outlier (MR-PRESSO).

**Results:**

In reverse MR results, a genetic prediction of short TL was causally associated with a higher risk of periodontitis (IVW: odds ratio [OR]: 1.0601, 95% confidence interval [CI]: 1.0213 to 1.1002; P =0.0021) and other complementary MR methods. In the forward MR analysis, periodontitis was shown to have no significant effect on TL (IVW: p = 0.7242), with consistent results for the remaining complementary MR. No pleiotropy was detected in sensitivity analysis (all P>0.05).

**Conclusion:**

Our MR studies showed a reverse causal relationship, with shorten TL being linked to a higher risk of periodontitis, rather than periodontitis shorten that TL. Future research is needed to investigate the relationship between cell senescence and the disease.

## 1 Introduction

Periodontitis is an inflammatory immune condition brought on by problems with mouth microbes (1). If periodontitis is not control in time, with the continuous development of inflammation, it may eventually destroy the tooth supporting tissue, leading to tooth loss (2, 3). Being a very common non-communicable disease, it has negatively impacted people’s quality of life and added to society’s financial burden (4).It is the sixth most common diseased in the globe. A person with many missing teeth may appear older, lose their ability to chew and have pronunciation issues. Periodontitis has significant systemic effects in addition to local ones, and prior studies have demonstrated a relationship between periodontitis and cardiovascular, respiratory, hypertensive, and diabetes disorders (5–7). Treatment of periodontitis is made more challenging by the interaction of multiple systemic diseases, and vice versa.

Under the accumulation of plaque, inflammation, and a bad lifestyle, the occurrence of periodontitis seems to be closely related to age (8). According to figures from epidemiological surveys, periodontitis affects 50% of adults in varying degrees, and incidence and severity have both sharply increased in the group over 65 (9). Periodontal attachment loss has been consistently observed to increase with age in various prospective cohort studies. However, the potentially nagetive impact of aging on periodontitis is still debatable, maybe as a result of the true risk factors of ongoing and cumulative periodontitis exposure (10, 11). This indicates that confounding and reverse causality may impair cohort studies and epidemiological research, making causal inference challenging. In conclusion, it is unclear at this time if age has a role in periodontitis risk.

The length of the telomere, which is a TTAGGG nucleotide repeat at the end of a chromosome that protects DNA and maintains chromosomal stability, can be used to measure the biological aging process (12, 13). Telomeres gradually shorten with each cell division due to the DNA replication mechanism’s inability to fully copy the 5’ end of the lagging DNA chain (14). This process eventually results in cell senescence, which can be accelerated by oxidative stress and inflammation.

Even though prior research on the mechanism of telomere erosion has demonstrated that inflammation plays a significant role in TL shortening (15). However, it is uncertain if periodontitis and TL shortening are connected. Previous research has found that individuals with periodontitis have lower TL values than non-periodontitis patients, and that measured TL values are associated with the severity of oxidative stress and periodontitis, but only in chronic instances (16). A recent study with 3,478 participants demonstrated a link between periodontitis and short TLs; However, cross-sectional studies cannot differentiate the two (17). Furthermore, the TL variation pattern is more consistent with a continuous dynamic fluctuation condition than a monotonous linear increase or drop (18). In observational studies, it may be challenging to control all confounding variables, which causes incorrect conclusions to be drawn about age and the relationship between TL and periodontitis. As a result, there are many conflicting findings about the relationship between periodontitis and TL.

The initial investigation in Mendelian randomization (MR) is genomic sequence analysis (19). Genetic information is difficult to misinterpret and independent of illness state, preventing reverse causation bias, hence it overcomes some limitations of observational investigations. The design of a random allocation trial is comparable to the natural random allocation that takes place during the development of each individual’s genetic makeup (20). The MR approach employs genetic markers as a tool variable (IV) for determining causality. Because confounders are frequently unrelated to genetic variation, disparities in outcomes between those who have the variant and those who do not can be attributed to differences in risk factors or susceptibility. As a result, unlike traditional observational studies, which are susceptible to confounding or reversing the causal relationship, MR gives a credible explanation for the varied exposure to the trait of interest (21). To present, no research has been undertaken employing MR approaches to explain the relationship between periodontitis and TL. We performed a bi-directional MR study and a series of sensitivity analyses to validate our hypothesis: (1) whether TL can be used as an effective means of identifying the occurrence of periodontitis. (2) whether shortened TL increases the susceptibility to periodontitis.

## 2 Material and method

### 2.1 Mendelian randomization

**Figure 1:**
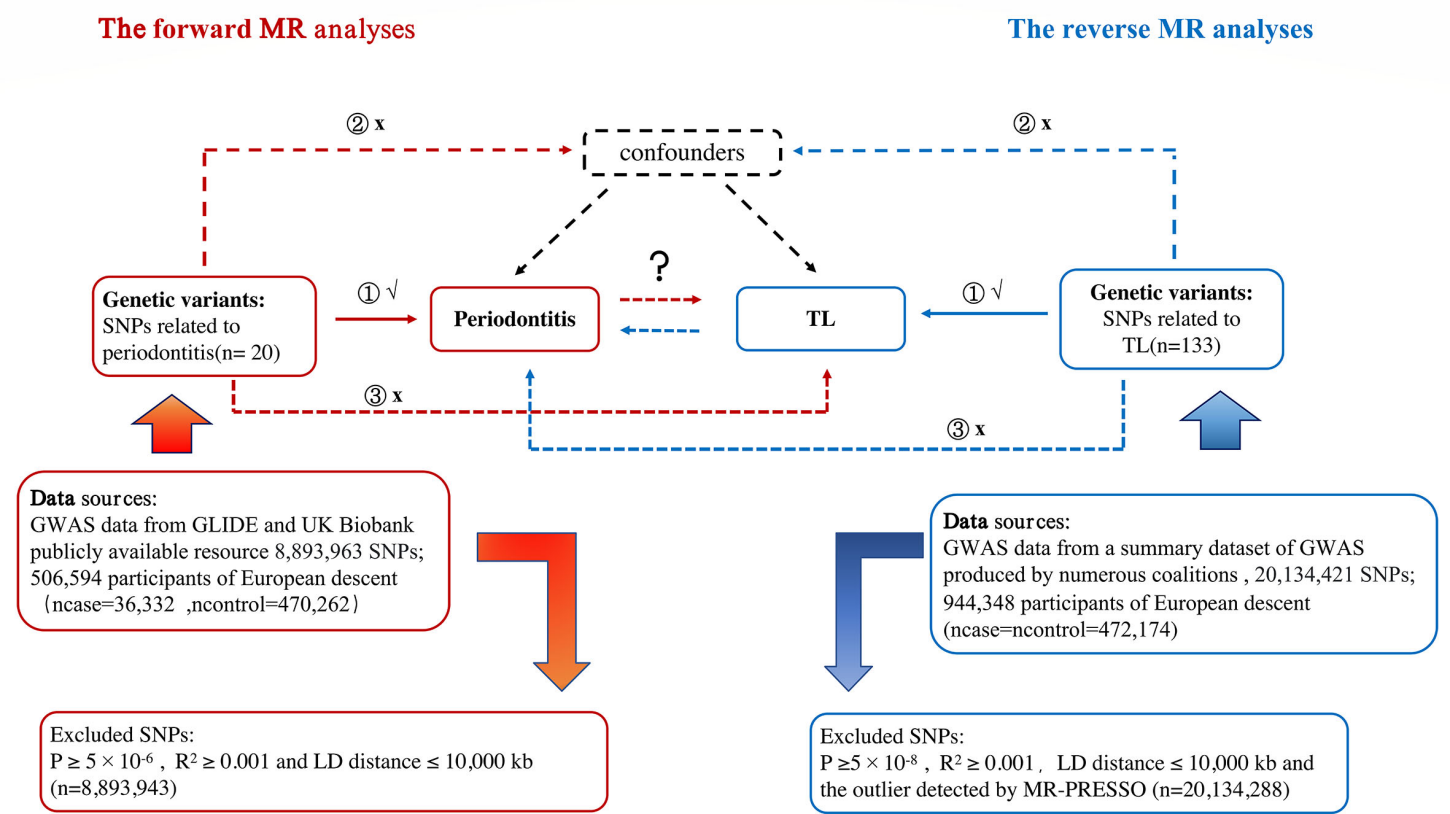
Study Design Sketch: Periodontitis is exposed in red in the forward MR analysis, and TL is the outcome. Exposure is represented by the color blue in the reverse MR analysis, periodontitis is the outcome. Abbreviation: TL, telomere length; MR, mendelian randomization; SNP, single nucleotide polymorphism.

To explore potential causal linkages between exposure and outcomes of interest, MR analyses will use genetic changes that are closely related to exposure as instrumental variables (IVs) (22). The MR estimate technique was unaffected by measurement mistakes, reverse causality, or confounding since genetic variations were randomly assigned at conception. A genetic variant must satisfy three essential conditions to be a legitimate IV (23) (1): There is a strong relationship between the instruments and exposure (“association”) (2); The tool influences outcomes through exposure (“exclusion limit”); (3) Genetic variables were unrelated to confounding variables of exposure outcomes (”exchangeability”); The bi-directional MR design flow between periodontitis and TL can be seen (**Figure 1**).

### 2.2 Data source

The largest meta-analyses, which included data from the UK Biobank (UKB) and the Gene-Lifestyle Interactions in Dental Endpoints (GLIDE) consortium, were used to create summary statistics for periodontitis (24). The SNPs were linked to the composite phenotype, which included self-reported loose teeth from UKB and clinically diagnosed periodontitis from GLIDE (Ncases = 18,979, NControls = 442,052). The Centers for Disease Control and Prevention (CDC)/American Academy of Periodontology definition(AAP), comparable standards assessed by probing depth, or self-reporting were used to categorize periodontitis cases (25). And 944,348 participants had TL phenotypic data from a summary dataset of GWAS produced by numerous coalitions (round 2, 2021), with a 1:1 case-control ratio. The GWAS summary data of TL were obtained from the IEU open GWAS project (https://gwas.mrcieu.ac.uk/). All the above populations are of European origin to minimize potential bias due to demographic heterogeneity.

### 2.3 Selection of the genetic instruments

At p<5×10^-8^ for TL as the significance threshold, we chose SNPs related to the exposure (26). To expand the statistical effect, the indicative association threshold for periodontitis was set to p < 5 × 10 ^− 6^. By calculating pair-wise linkage disequilibrium and excluding SNPs with r^2^ ≥0.001 and LD distance ≤ 10,000 kb from this set of SNPs, we chose only independent instruments with the lowest p-value (27). The flowchart of the study is presented in **Figure 1**. F statistic needs to be higher than 10 to get enough strength to limit the deviation from weak tool variables (28). F to calculate statistics, we use: [(R^2^× (N-2))/(1-R^2^)].

The impacts of several genetic variations were coordinated for the analysis, and SNPs made up of one base and its complementary base were disregarded. We only used variations appropriate for all observed traits and did not employ stand-ins for the missing variants to preserve the consistency of SNPs used as IVs in various analyses (23).

### 2.4 Statistical analyses

Six different MR inverse variance weighting (IVW) of random effects, MR Egger, Weighted median, Simple mode, MR-PRESSO and Weighted mode approaches were performed to address variability heterogeneity and pleiotropic effects (29). MR analysis was repeated if significant horizontal pleiotropy was detected in the MR-PRESSO analysis (with a P-value lower than the threshold in the MR-PRESSO outlier test). The inverse variance weighted (IVW) model is used as the main analysis, because it is a meta-method, which combines the Wald estimation of each SNP and effectively regards each SNP as an effective natural experiment. Importantly, it forces the intercept in the regression slope to be zero, so if any IV is invalid, the result may be biased (30). Then MR-Egger and weighted medians were used to enhance IVW estimates since they could offer a more accurate estimate in a wider range of circumstances, although being less efficient (broader CIs) (31). While pleiotropy is permitted for all genetic variations under MR-Egger, it must be independent of variation exposure (32). At the Same Time, the simple and weighted modes were used to evaluate the robustness of the IVW method results. Finally, the MR-Egger intercept test and leave-one-out analysis were used to evaluate horizontal pleiotropy further. Heterogeneity was also identified using Cochran’s Q test and I^2^. I^2^ statistics may indicate dilution in MR-Egger estimates, which may mean that MR-Egger results may be inaccurate. Several plots were used to evaluate the significant SNPs (leave-one-out, funnel plot, forest plot, and scatter plot) (33).

The study made use of de-identified data from participating studies that were made publically accessible and had their use of human subjects approved by the ethical standards board. This study doesn’t need any additional ethical approval.

The R (version 3.6.1) package’s Two Sample MR (version 0.4.25) and MR-PRESSO (version 1.0) packages were used for all studies.

## 3 Results

### 3.1 Effect of periodontitis on TL

After screening, 20 independent SNPs related to periodontitis were identified. The minimum f statistics of these IV’s are all greater than 10 (ranging from 89 to 185), which ensures the hypothesis of “correlation”, that is, weak instrument bias is unlikely to affect the estimation of causal effects. Meanwhile, no pleiotropy was found based on Egger intercept (intercept =0.0016, P =0.401), **Table 1**; And MR-PRESSO did not detect heterogeneity (Q =15.7, I^2 =^ 0.11, P =0.330), **Table 2**.

**Table 1:** MR-Egger test for directional pleiotropy.

exposure	outcome	intercept	SE	p-value
Periodontitis	TL	0.0016	0.0019	0.401
TL	periodontitis	0.0006	0.0009	0.532

df, degree of freedom; MR, Mendelian randomization; Q, heterogeneity statistic Q.

**Table 2:** Heterogeneity of wald ratios.

exposure	outcome	Q	df	I^2^	p-value
periodontitis	TL	15.7	14	0.11	0.330
TL	periodontitis	130.3	113	0.13	0.120

df, degree of freedom; MR, Mendelian randomization; Q, heterogeneity statistic Q.

**Figure 2:**
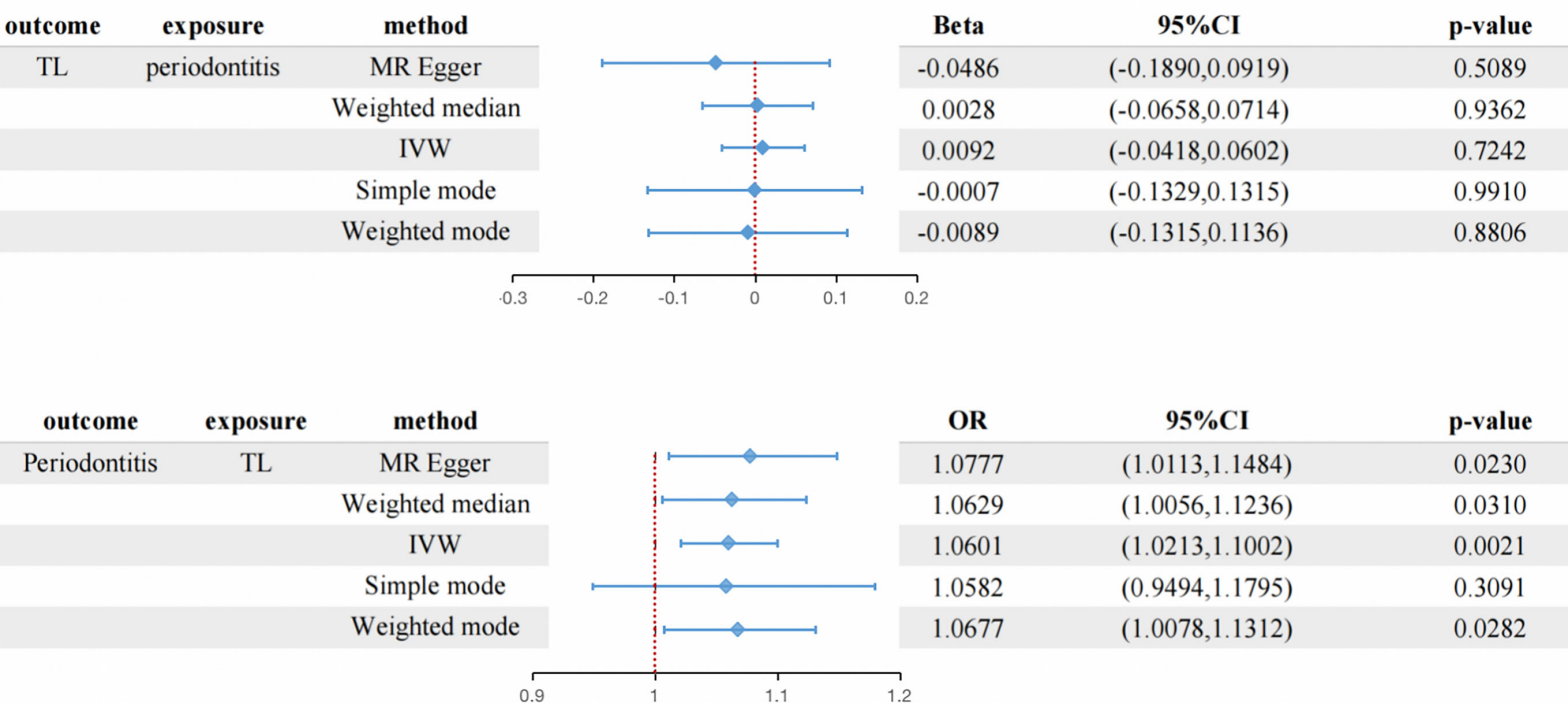
The connection between genetically instrumented periodontitis and TL, and vice versa is estimated using MR(the table at the bottom shows the OR of TL shortening to periodontitis) CI, confidence interval; IVW, inverse variance-weighted; MR, Mendelian randomization; OR, odds ratio.

The MR estimation values of different methods are listed in **Figure 2**. Overall, in the main result IVW, there is no causal relationship between the genetically predicted periodontitis and TL (beta=0.0092, 95% CI:-0.0418 to 0.0602, P = 0.7242). In addition, MR Egger, weighted median, weighted mode method and simple mode method show consistent results. The scatter plot of SNP effect of periodontitis and TL shows the **Supplementary Graph S1**. According to the heterogeneity test, there is no heterogeneity among individual SNPs. One-way analysis showed that the causal estimation of periodontitis was not driven by any single SNP. One-way analysis diagram, forest diagram and funnel diagram are shown in **Supplementary Diagrams S2-S4**.

### 3.2 Effect of TL on periodontitis

After the initial screening, 134 independent SNPs associated with TL were identified. The minimum F statistics of these IV are all greater than 10 (ranging from 57 to 3086), which ensures the hypothesis of “correlation”, that is, weak instrumental bias is unlikely to affect the estimation of causal effect. However, MR-PRESSO found that rs10773176 was an extremely abnormal variation. After removing this outlier, heterogeneity is no longer detected (Q =130.3,I^2 =^ 0.13^ P^ =0.120), **Table 2**; At the same time, no pleiotropy was found based on Egger intercept (intercept=0.0006, P =0.532), **Table 1**. So the finally determined effective IVs are 133 SNPs. The characteristics of all SNPs included in IVs as TL are detailed in **Supplementary Table 2**.

**Figure 3:**
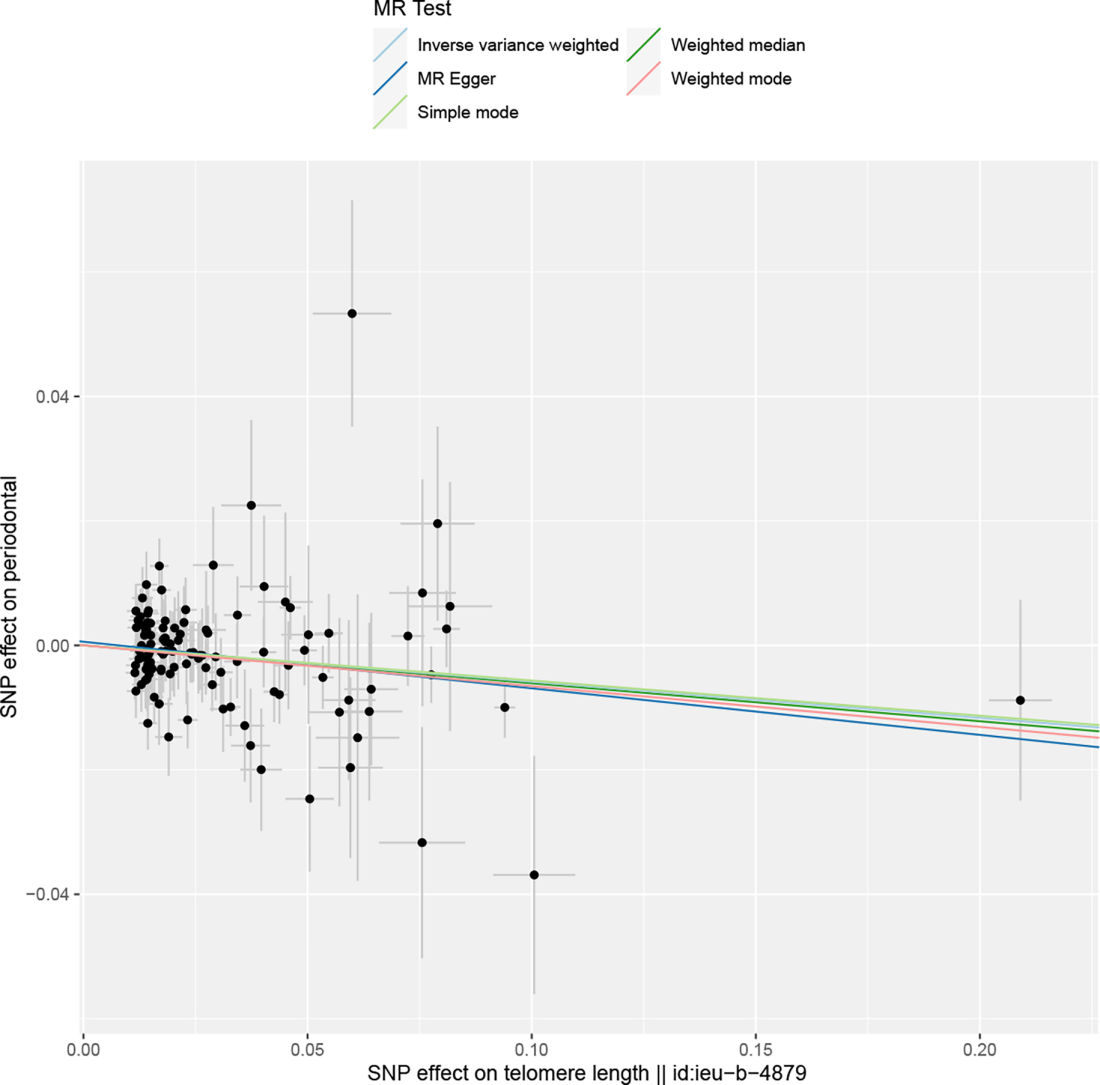
The reverse MR: Scatter plot of the effects of SNPs on TL and periodontitis. The horizontal and vertical axes represent the effect of each genetic variation on telomere length and periodontitis. The gray line around the black solid point indicates the corresponding 95% CI for the effect. The slopes of the solid lines show the effect estimates of the five MR methods. MR, mendelian randomization; SNP, single nucleotide polymorphism; CI, confidence interval.

**Figure 4:**
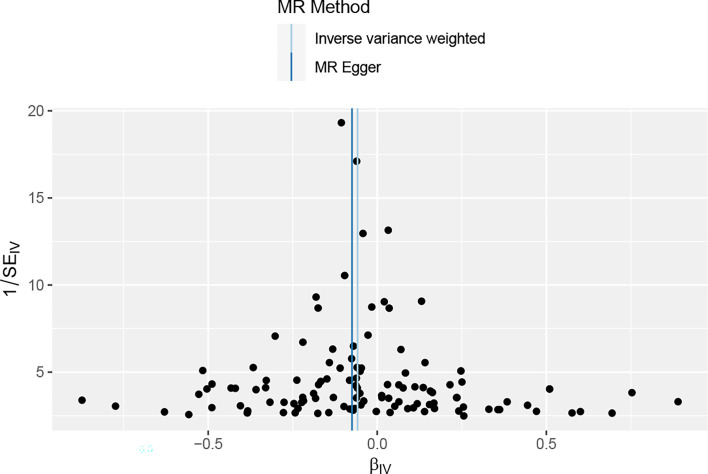
The funnel plot of the association between periodontal and TL, reverse MR analysis;It is used to determine whether the associations observed are noticeably heterogeneous.

Overall, in the main analysis using IVW combined with multiple genetic variations, shorter TL has a significant causal relationship with higher odds of periodontitis (OR: 1.0601, 95% CI: 1.0213 to 1.1002, P = 0.0021). For every standard deviation decrease of TL, The odds ratio of periodontitis was 1.0601 (**Figure 2**). The weighted median (OR: 1.0629, 95% CI: 1.0056-1.1236, P = 0.0310), MR-Egger (OR:1.0677, 95% CI: 1.0113-1.10) were used. 95% CI: 1.0078-1.1312, P =0.0282) The methods have the same direction, and have comparable point estimates and confidence intervals. These causal estimates are further shown in the scatter plot and the funnel plot (**Figures 3**, **4**).

One-way analysis diagram and forest diagram are shown in **Supplementary Diagrams S5-S6**.

## 4 Discussion

In this study, we used the largest GWAS summary-level data set accessible to date to conduct a two-sample MR analysis to completely evaluate the causal effect of periodontitis on TL. This is the first study to investigate the bidirectional causal relationship between periodontitis and TL by performing multiple complementary MR approaches. No evidence of periodontitis and TL associations supporting genetic prediction was observed in our two-sample MR forward analysis. However, reverse MR analysis showed evidence that TL were related to periodontitis, and shortened TL increased the risk of periodontitis.

Various studies have examined whether there is a link between TL and periodontitis in recent years (34). According to one theory, systemic inflammation and oxidative stress brought on by chronic periodontitis may shortened TL, as evidenced by previous case-control studies (16, 35). At the same time, consistent results from two large cross-sectional studies conducted based on the National Health and Nutrition Examination Survey (NHANES) confirmed the association of periodontitis with TL (17, 36). The study examined the depth of probing, loss of adhesion, and probing bleeding from the distal, mesial, or midfacial region of each tooth in two randomly selected quadrants. And the severity of periodontitis was defined according to the CDC/AAP Sciences guidelines to ensure a thorough periodontal evaluation. However, due to the drawbacks of cross-sectional studies, it is still unclear whether periodontitis causes shortened TL or whether shortened TL increases susceptibility to periodontitis, even if there is a potential inflammatory mechanism to explain the effect of periodontitis on TL.

Although different results have been obtained in the past two prospective studies that there was no significant association between TL and periodontitis (10, 37). This result may be limited by several reasons, such as a small study span, younger age of the covered population, and a periodontal examination result without full mouth examination (38). Most importantly, insufficient numbers of people are likely to result in insufficient statistical capacity. Researchers have long questioned whether populations with shortened TL are more prone to periodontitis because a reverse causal relationship cannot be ruled out, even if a cross-sectional study confirms an association between the two (36). However, ethical and moral limitations make large-scale randomized controlled trial (RCT) research challenging. To fully reveal these causal relationships, it is more practical to collect evidence using the MR method. We investigated the potential causal effects of periodontitis on TL using MR methods.

The following possible mechanisms underlie the reverse causal relationship that we found in MR studies between periodontitis and shortened TL. First, shortened TL have been found to affect the proliferation, migration, and mesodermal lineage differentiation of periodontal ligament stem cells, and these properties are essential for periodontal tissue regeneration in periodontitis (39). Another explanation may be that telomere dysfunction can activate the production and secretion of inflammatory factors such as IL-6 and TNF-α (40), leading to the development of periodontitis. Additionally, many cellular functions start to malfunction as TL decrease (34). Therefore, in people with periodontitis, immune cells with shorten telomeres may indicate immune system dysfunction, encourage the growth of local gingival bacteria, and favor the development of oral diseases. This is probably a potential mechanism by which we arrived at this conclusion, and more research is required to determine the mechanism by which shortened telomeres affect periodontitis in the future.

It is worth noting that physiological age is often more accurate than chronological age at predicting healthy aging and is clinically more relevant (41). TL is regarded as a reliable indicator of biological age because telomeres have a significant impact on the rate of cell senescence and death. Our results showed that a shorter TL may increase the odds of periodontitis, but it is likely to be unrelated to actual age (42). At present, the correlation between TL and physiological age has been confirmed by many studies, and it is found that physiological age is affected by many lifestyle factors, including diet, smoking, exercise and sleep habits, which are also found to be related to the disease factors of periodontitis (43). And other illnesses linked to shorter telomeres, like as atherosclerosis, diabetes and obesity, have also been linked to periodontitis (24, 44). Based on our research results, whether TL plays an intermediary role is a potential direction for future research (45).

Our current findings suggest that TL may be a targetable factor in the development of new preventive measures to address periodontitis risk. Although practical interventions that directly alter TL are not yet available (46), they may be possible through a healthy lifestyle (47). Reverse MR supports the view that shorter telomeres may lead to a higher risk of periodontitis, but the clinical significance of MR estimation should be interpreted with caution (23). It must be pointed out that, as with all other MR investigations, it should be better interpreted as test statistics for causal hypotheses, providing alternative etiological evidence supporting a causal relationship between TL and periodontitis.

Our research offers several significant advantages. The large sample size of the two-sample MR study design, as well as the inclusion of genetically predicted phenotypes as exposures of interest in MR studies, decreases the potential for reverse causation and confounding bias when compared to observational studies. Furthermore, the study was developed with only the European population in mind, avoiding aberrations owing to demographic variability.

Our study has the following limitations. First, while our study avoided population abnormality by controlling for the participants’ ethnicity, whether our findings were universal for other groups remains to be determined. Second, due to the low heritability of periodontitis, the estimation of the genetic relationship between TL and periodontitis may be skewed. However, because TL is impacted by hereditary factors, the effect of bias on the results should be insignificant. Furthermore, the periodontitis GWAS data we used did not stratify disease severity (48). As a result, when more thorough GWAS data becomes available in the future, more studies will be required to validate our findings.

## 5 Conclusion

Periodontitis did not significantly affect TL in our forward MR study, but in reverse MR, we discovered an inverse causal relationship between the two, meaning that a shortened TL is linked to a higher risk of developing periodontitis. Further investigation into the connection between cell senescence and periodontitis as well as validation of TL as a biomarker for predicting the development of periodontitis are necessary given that this may suggest that telomere biology is a potential pathway involved in the occurrence and development of periodontitis.

## Data availability statement

The original contributions presented in the study are included in the article/**Supplementary Material**. Further inquiries can be directed to the corresponding authors.

## Author contributions

JH designed the research, contributed to data interpretation, and drafted the manuscript. ZC, JY, FJ, QP, XC, QS and YT contributed to data interpretation and the manuscript. YL, LC, and JS reviewed this article. JH and JS contributed equally to this work and should be considered co-first authors. YL and LC contributed equally to this work and share senior authorship. All authors read and approved the final manuscript. All authors have read and agreed to the published version of the manuscript.All authors contributed to the article and approved the submitted version.
