# Supplementary material for: Reverse causal relationship between periodontitis and shortened telomere length: Bidirectional two-sample Mendelian random analysis
Source: Front Immunol. 2022 Dec 19;13:1057602. doi: 10.3389/fimmu.2022.1057602 (PMC9806346; doi:10.3389/fimmu.2022.1057602)
Supplement: Supplementary file 1 [file DataSheet_1.pdf]

Supplementary table 1:Characteristics of SNPs selected as instrumental variables for Mendelian Randomization analysis(Exposure=Periodontitis，outcome= Telomere length)

Instrumental SNPs	Effect allele	Exposure			Outcome			R^2	F
		Beta	SE	P-value	P-value	SE	Beta		
rs112176734	C	0.406	0.087	3.30E-06	0.4	0.006	-0.005	0.009	1838.374
rs139232605	A	0.226	0.049	3.40E-06	0.94	0.007	0.000	0.002	474.073
rs140906408	C	0.179	0.038	1.99E-06	0.85	0.004	0.001	0.005	1052.314
rs143322523	C	-0.244	0.052	2.61E-06	0.88	0.006	0.001	0.005	924.031
rs148828254	G	0.309	0.067	4.28E-06	0.25	0.011	-0.012	0.002	364.871
rs4880548	G	0.130	0.028	3.78E-06	0.22	0.002	0.003	0.007	1348.866
rs6924687	T	0.144	0.031	2.55E-06	0.78	0.003	-0.001	0.006	1248.625
rs79531174	T	0.321	0.070	4.78E-06	0.14	0.010	-0.014	0.002	441.663

Supplementary table 2:Characteristics of SNPs selected as instrumental variables for Mendelian Randomization analysis(Exposure=Telomere length，outcome=Periodontitis)

Instrumental SNPs	Effect allele	Exposure			Outcome			R^2	F
		Beta	SE	P-value	Beta	SE	P-value		
rs1003322	A	0.0142	0.0025	1.00E-08	0.0237	0.0359	0.5092	0.0001	63.9971
rs10112752	A	-0.0288	0.0020	9.50E-46	0.0263	0.0254	0.3020	0.0004	384.2068
rs1023767	A	-0.0184	0.0023	5.00E-15	-0.0030	0.0261	0.9078	0.0001	115.8456
rs10768683	G	0.0470	0.0028	1.50E-64	0.0413	0.0315	0.1895	0.0006	558.2216
rs10773176	G	-0.0172	0.0023	5.20E-14	-0.0078	0.0319	0.8076	0.0001	107.1931
rs10774624	A	0.0150	0.0021	2.90E-13	0.0114	0.0258	0.6601	0.0001	105.7936
rs10805346	C	0.0117	0.0020	7.00E-09	0.0270	0.0255	0.2906	0.0001	63.6875
rs10840270	G	0.0144	0.0021	1.30E-11	-0.0195	0.0269	0.4693	0.0001	88.4238
rs10845387	A	-0.0141	0.0021	1.50E-11	-0.0172	0.0273	0.5286	0.0001	85.7334
rs10905255	T	-0.0182	0.0020	2.60E-19	0.0133	0.0255	0.6030	0.0002	152.5030
rs11085072	T	-0.0132	0.0024	2.60E-08	-0.0016	0.0342	0.9616	0.0001	59.4953
rs11117354	C	0.0233	0.0022	3.40E-26	0.0136	0.0256	0.5959	0.0002	216.7966
rs111527438	C	0.0125	0.0021	3.10E-09	0.0345	0.0271	0.2029	0.0001	67.2565
rs111950327	C	0.0238	0.0041	5.90E-09	-0.0399	0.0567	0.4820	0.0001	63.7181
rs112394943	C	-0.0199	0.0028	1.60E-12	0.0645	0.0302	0.0327	0.0001	101.9020
rs113525195	A	-0.0124	0.0022	3.10E-08	-0.0191	0.0275	0.4885	0.0001	59.8348
rs11557154	T	-0.0344	0.0030	1.10E-30	0.0151	0.0372	0.6843	0.0003	252.8465
rs11579626	C	0.0265	0.0036	1.30E-13	0.0772	0.0387	0.0463	0.0001	103.0568
rs11584821	T	-0.0307	0.0026	3.00E-31	0.0366	0.0329	0.2656	0.0003	258.4547
rs116863223	A	-0.0818	0.0094	2.60E-18	-0.0842	0.1012	0.4058	0.0002	147.3881
rs11699829	A	0.0642	0.0060	1.50E-26	0.0724	0.1037	0.4855	0.0003	256.4695

rs117407747	T	0.0451	0.0061	1.80E-13	-0.0115	0.1334	0.9311	0.0001	103.1135
rs117512405	A	-0.0790	0.0082	9.50E-22	-0.0616	0.0569	0.2790	0.0002	197.0191
rs117630647	A	0.0596	0.0072	1.40E-16	0.0112	0.0987	0.9096	0.0001	139.8773
rs11769630	A	-0.0257	0.0039	4.30E-11	0.0314	0.0415	0.4494	0.0001	83.5713
rs11991877	A	-0.0301	0.0032	3.20E-21	0.0092	0.0361	0.7976	0.0002	168.4874
rs12369950	C	-0.0178	0.0029	8.00E-10	-0.0261	0.0356	0.4626	0.0001	72.3558
rs12412214	A	-0.0245	0.0022	3.40E-28	-0.0100	0.0259	0.6995	0.0002	228.5068
rs12451892	C	-0.0116	0.0021	2.20E-08	-0.0034	0.0257	0.8958	0.0001	59.9102
rs1291143	C	0.0493	0.0028	1.80E-69	-0.0123	0.0304	0.6863	0.0006	588.8577
rs12925933	C	-0.0147	0.0021	7.00E-12	-0.0220	0.0254	0.3873	0.0001	91.3033
rs12932179	G	-0.0136	0.0020	1.80E-11	0.0162	0.0260	0.5332	0.0001	86.0240
rs13062095	C	0.0139	0.0021	9.70E-11	0.0418	0.0261	0.1092	0.0001	80.4147
rs13230646	C	-0.0173	0.0023	8.90E-14	-0.0009	0.0333	0.9795	0.0001	105.6877
rs1332941	G	0.0257	0.0027	5.90E-21	0.0337	0.0312	0.2808	0.0002	183.7617
rs137901416	A	0.0457	0.0033	4.70E-43	0.0222	0.0511	0.6647	0.0004	356.0868
rs139669835	T	-0.0613	0.0105	6.10E-09	-0.3643	0.3392	0.2828	0.0001	66.0904
rs139795227	C	0.0599	0.0087	6.70E-12	-0.0803	0.0801	0.3160	0.0001	93.5541
rs141214782	TTATC	-0.0247	0.0034	2.00E-13	0.0201	0.0410	0.6238	0.0001	104.8206
rs142426306	T	-0.0505	0.0054	8.70E-21	0.0875	0.0645	0.1749	0.0002	182.7774
rs143190905	T	-0.0724	0.0037	1.60E-85	-0.0807	0.0478	0.0914	0.0008	732.5378
rs144204502	T	-0.1006	0.0091	3.40E-28	0.0187	0.0671	0.7811	0.0003	237.8648
rs145114957	G	0.0273	0.0050	4.60E-08	0.0898	0.0649	0.1661	0.0001	57.4137
rs150150565	T	0.0638	0.0074	6.80E-18	-0.0763	0.0937	0.4155	0.0002	161.7619
rs1611236	A	-0.0160	0.0021	6.10E-14	0.0212	0.0303	0.4839	0.0001	106.4007
rs16978028	T	-0.0299	0.0029	8.20E-26	0.0358	0.0461	0.4369	0.0002	207.8173
rs17445108	A	-0.0169	0.0030	2.00E-08	-0.0141	0.0386	0.7159	0.0001	59.8109
rs17677991	G	0.0223	0.0021	4.40E-26	-0.0205	0.0258	0.4274	0.0002	211.4370
rs182059586	C	-0.0571	0.0068	4.90E-17	-0.1020	0.1536	0.5066	0.0002	150.7081
rs185174247	A	0.0373	0.0044	1.10E-17	0.0268	0.0738	0.7165	0.0001	139.1655
rs188918174	T	0.0403	0.0054	1.20E-13	-0.0097	0.0597	0.8714	0.0001	106.7479
rs1907702	A	0.0150	0.0024	5.90E-10	-0.0691	0.0316	0.0290	0.0001	75.9957
rs1957937	T	0.0209	0.0027	1.90E-14	0.0560	0.0325	0.0844	0.0001	111.0052
rs1985369	G	-0.0312	0.0030	3.60E-25	0.0261	0.0407	0.5215	0.0002	210.4274
rs2056726	A	-0.0228	0.0024	7.90E-21	0.0219	0.0302	0.4680	0.0002	165.3991
rs2230590	C	-0.0158	0.0020	3.60E-15	0.0311	0.0257	0.2270	0.0001	117.8320
rs2276182	G	0.0234	0.0020	2.80E-30	0.0098	0.0254	0.7002	0.0003	248.9182
rs2282764	G	-0.0224	0.0029	9.30E-15	-0.0503	0.0361	0.1636	0.0001	115.7458
rs2293579	A	-0.0129	0.0021	3.30E-10	0.0254	0.0276	0.3573	0.0001	74.5171
rs2306646	C	-0.0209	0.0020	3.30E-25	0.0112	0.0253	0.6585	0.0002	203.3730

rs2538745	C	-0.0129	0.0021	3.10E-10	-0.0205	0.0253	0.4185	0.0001	75.2589
rs2555104	C	-0.0140	0.0020	6.60E-12	0.0139	0.0252	0.5806	0.0001	90.9568
rs2763979	T	-0.0278	0.0021	1.30E-40	-0.0348	0.0254	0.1707	0.0004	336.3019
rs28363070	A	0.0756	0.0096	3.50E-15	-0.1368	0.2847	0.6308	0.0002	142.7303
rs28502153	A	-0.0216	0.0021	1.20E-25	-0.0269	0.0260	0.3004	0.0002	207.2269
rs28577594	C	0.0188	0.0022	5.40E-17	-0.0253	0.0281	0.3664	0.0001	137.5224
rs2967355	C	-0.0462	0.0024	4.00E-83	0.0477	0.0332	0.1507	0.0007	705.0339
rs2977608	C	0.0129	0.0023	3.00E-08	0.0265	0.0275	0.3361	0.0001	59.8814
rs3093888	A	-0.0290	0.0045	1.50E-10	-0.0233	0.0442	0.5985	0.0001	77.3106
rs35446936	A	-0.0940	0.0023	1.00E-200	-0.0482	0.0284	0.0889	0.0033	3085.9106
rs35640778	A	-0.2090	0.0070	9.59E-195	-0.0380	0.1137	0.7385	0.0018	1683.3014
rs3767952	A	0.0134	0.0024	1.80E-08	-0.0293	0.0303	0.3334	0.0001	59.4563
rs3785074	G	0.0239	0.0022	2.60E-27	-0.0059	0.0310	0.8489	0.0002	222.0493
rs3891167	G	-0.0426	0.0024	1.20E-70	-0.0017	0.0295	0.9538	0.0007	648.8932
rs41269079	A	0.0154	0.0025	1.70E-09	-0.0406	0.0310	0.1904	0.0001	68.6621
rs41304832	A	0.0612	0.0093	5.00E-11	0.0228	0.0663	0.7311	0.0001	86.6376
rs429358	C	0.0173	0.0028	3.80E-10	0.0417	0.0330	0.2071	0.0001	73.6509
rs4498805	T	0.0151	0.0020	5.70E-14	-0.0089	0.0252	0.7238	0.0001	106.7371
rs4530278	T	0.0139	0.0021	1.50E-11	0.0080	0.0258	0.7581	0.0001	87.7177
rs45604339	T	-0.0204	0.0021	4.30E-22	-0.0254	0.0260	0.3273	0.0002	177.0103
rs4616688	T	-0.0173	0.0020	4.50E-18	0.0105	0.0258	0.6833	0.0001	140.9730
rs4695407	G	0.0142	0.0020	1.50E-12	0.0125	0.0252	0.6193	0.0001	95.1954
rs4724	A	-0.0547	0.0031	9.80E-69	-0.0111	0.0410	0.7866	0.0006	582.4512
rs4731541	G	-0.0206	0.0021	1.40E-23	-0.0545	0.0262	0.0375	0.0002	187.9055
rs4743037	T	0.0148	0.0024	5.10E-10	-0.0313	0.0306	0.3055	0.0001	73.4725
rs55747751	A	-0.0212	0.0038	1.70E-08	0.0529	0.0549	0.3359	0.0001	60.6199
rs56178008	A	0.0144	0.0020	9.70E-13	0.0484	0.0255	0.0578	0.0001	96.3898
rs56799554	G	-0.0260	0.0027	3.00E-22	0.0313	0.0302	0.2990	0.0002	180.3532
rs5742915	C	0.0193	0.0020	1.60E-21	0.0517	0.0256	0.0436	0.0002	173.8450
rs59409453	G	0.0202	0.0023	1.60E-18	-0.0180	0.0312	0.5642	0.0002	151.7088
rs6007020	C	0.0145	0.0021	4.80E-12	0.0334	0.0264	0.2065	0.0001	92.3434
rs6054257	A	-0.0142	0.0025	1.10E-08	-0.0195	0.0317	0.5392	0.0001	62.4070
rs611646	A	-0.0368	0.0020	3.50E-73	0.0535	0.0258	0.0380	0.0007	618.5199
rs61405042	T	-0.0502	0.0060	8.50E-17	-0.0777	0.1411	0.5816	0.0001	135.3890
rs61748181	T	-0.0592	0.0060	2.80E-23	0.0983	0.0561	0.0797	0.0002	185.8026
rs6536702	A	0.0534	0.0024	9.40E-111	-0.0208	0.0325	0.5212	0.0010	941.2559
rs6584579	G	0.0115	0.0020	2.00E-08	-0.0367	0.0269	0.1730	0.0001	59.8956
rs6587577	G	-0.0182	0.0026	4.80E-12	0.0624	0.0346	0.0711	0.0001	89.8013
rs6659669	T	-0.0117	0.0021	1.10E-08	-0.0246	0.0255	0.3343	0.0001	61.7839

rs6669563	A	0.0182	0.0020	2.10E-19	-0.0004	0.0254	0.9866	0.0002	154.0073
rs66731853	A	-0.0178	0.0022	1.50E-16	-0.0099	0.0318	0.7568	0.0001	129.6465
rs670180	A	-0.0116	0.0020	1.20E-08	-0.0298	0.0254	0.2394	0.0001	62.3262
rs6751209	C	-0.0140	0.0025	1.60E-08	0.0294	0.0328	0.3688	0.0001	60.1595
rs6776756	A	-0.0174	0.0020	1.10E-17	0.0079	0.0256	0.7578	0.0001	137.5281
rs6790988	G	0.0146	0.0023	1.80E-10	0.0485	0.0300	0.1059	0.0001	77.0967
rs6881568	A	0.0169	0.0021	3.70E-16	-0.0465	0.0283	0.1003	0.0001	124.6900
rs7099229	A	-0.0153	0.0022	8.40E-12	-0.0150	0.0331	0.6498	0.0001	87.8171
rs7164950	G	0.0129	0.0020	2.30E-10	0.0039	0.0256	0.8781	0.0001	75.8033
rs7209057	A	0.0118	0.0020	5.70E-09	-0.0058	0.0254	0.8182	0.0001	64.7713
rs7221585	T	0.0143	0.0025	6.70E-09	-0.0146	0.0282	0.6049	0.0001	67.1388
rs73581419	T	0.0230	0.0032	1.30E-12	0.0105	0.0459	0.8190	0.0001	95.1620
rs73730598	A	0.0274	0.0044	4.70E-10	0.0007	0.0505	0.9889	0.0001	73.4514
rs75664430	G	-0.0235	0.0023	3.60E-24	-0.0395	0.0321	0.2186	0.0002	194.5610
rs76065543	T	0.0343	0.0029	4.20E-32	-0.0257	0.0328	0.4340	0.0003	263.5921
rs76219171	A	0.0360	0.0043	7.80E-17	0.0825	0.0678	0.2235	0.0001	134.6193
rs762679	A	0.0310	0.0029	1.40E-27	0.0105	0.0335	0.7532	0.0002	223.1343
rs76666449	C	0.0295	0.0033	8.20E-19	0.0235	0.0367	0.5215	0.0002	148.7389
rs7705526	A	0.0776	0.0022	1.00E-200	-0.0731	0.0268	0.0064	0.0026	2507.9897
rs77231040	C	0.0989	0.0135	2.00E-13	0.0037	0.0799	0.9631	0.0001	104.7114
rs7772289	T	0.0175	0.0020	1.70E-18	0.0163	0.0259	0.5301	0.0002	144.6196
rs77732866	A	0.0178	0.0029	9.20E-10	-0.0306	0.0364	0.4005	0.0001	71.0168
rs7790856	T	-0.0437	0.0022	1.80E-87	0.0234	0.0302	0.4382	0.0008	741.8595
rs78491606	C	-0.0756	0.0074	1.90E-24	0.1291	0.0785	0.1001	0.0002	195.0055
rs79977579	A	0.0282	0.0034	2.30E-16	0.0422	0.0430	0.3260	0.0001	129.8786
rs80116508	A	-0.0353	0.0042	2.00E-17	0.0273	0.0468	0.5599	0.0001	137.7134
rs80324517	A	0.0397	0.0047	1.80E-17	0.0684	0.0518	0.1869	0.0001	136.8524
rs8102497	A	-0.0150	0.0020	1.40E-13	0.0090	0.0254	0.7222	0.0001	104.2739
rs8105767	G	0.0328	0.0022	2.50E-50	0.0002	0.0271	0.9955	0.0004	422.5296
rs869785	C	-0.0147	0.0021	4.40E-12	-0.0110	0.0267	0.6798	0.0001	89.8961
rs871134	T	-0.0183	0.0020	1.70E-19	-0.0045	0.0254	0.8596	0.0002	155.1401
rs932002	T	-0.0402	0.0028	7.30E-47	-0.0120	0.0298	0.6859	0.0004	391.0249
rs9398196	G	-0.0144	0.0020	9.50E-13	0.0038	0.0261	0.8845	0.0001	97.7617
rs939916	A	0.0242	0.0022	6.60E-29	0.0129	0.0286	0.6523	0.0003	244.6206
rs9419958	C	-0.0810	0.0029	2.60E-167	0.0140	0.0394	0.7217	0.0016	1481.7679
rs9600019	T	0.0127	0.0021	2.40E-09	0.0180	0.0261	0.4902	0.0001	67.9284
rs9878436	T	-0.0143	0.0020	1.20E-12	0.0148	0.0264	0.5738	0.0001	94.9022
rs9940099	C	-0.0336	0.0041	3.20E-16	-0.0128	0.1262	0.9195	0.0001	125.3267
rs9955360	A	-0.0190	0.0030	2.20E-10	-0.0422	0.0361	0.2432	0.0001	77.4728

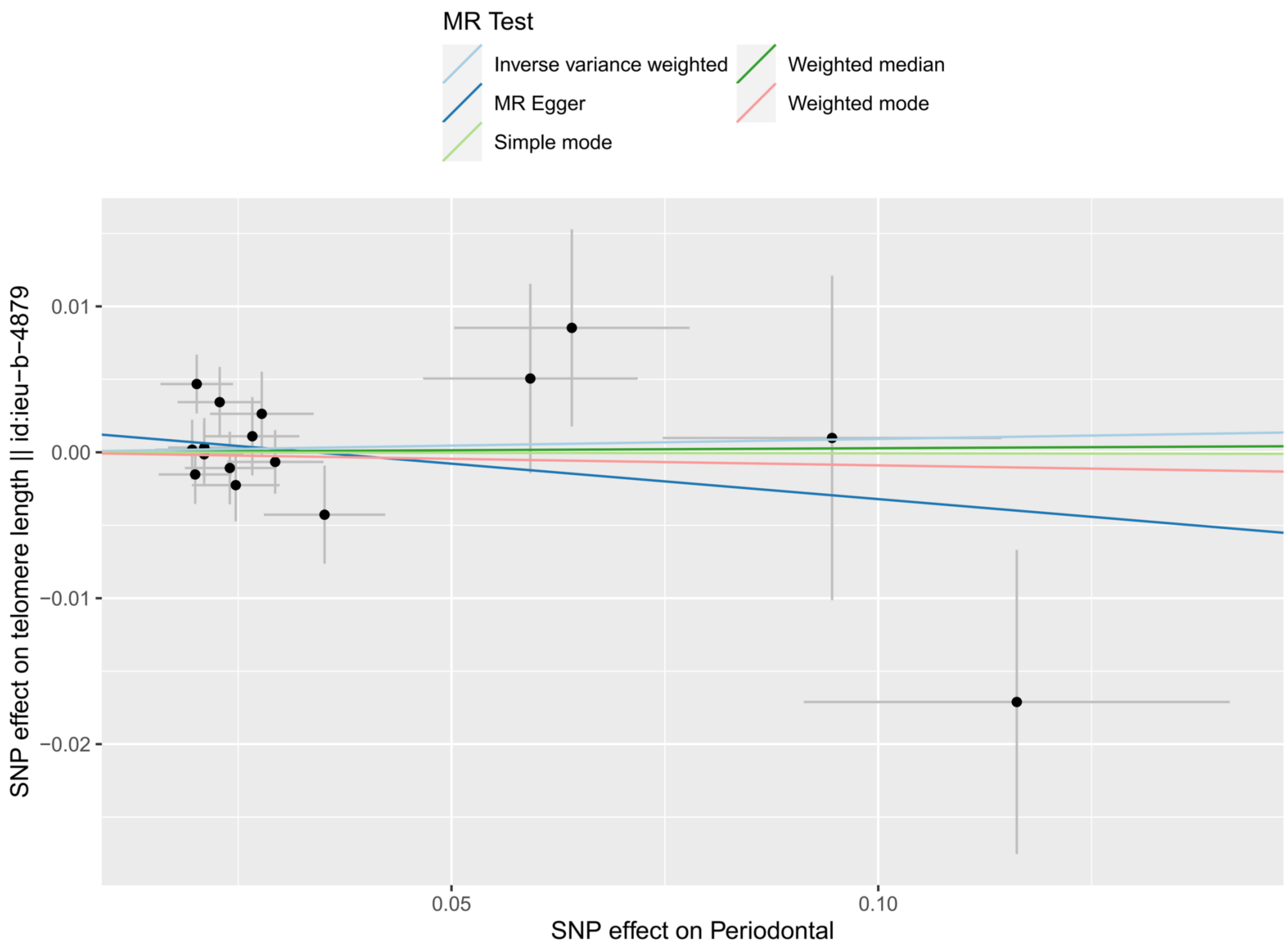

Supplementary figure 1, The forward MR Scatter plot of the effects of SNPs on TL and periodontitis,

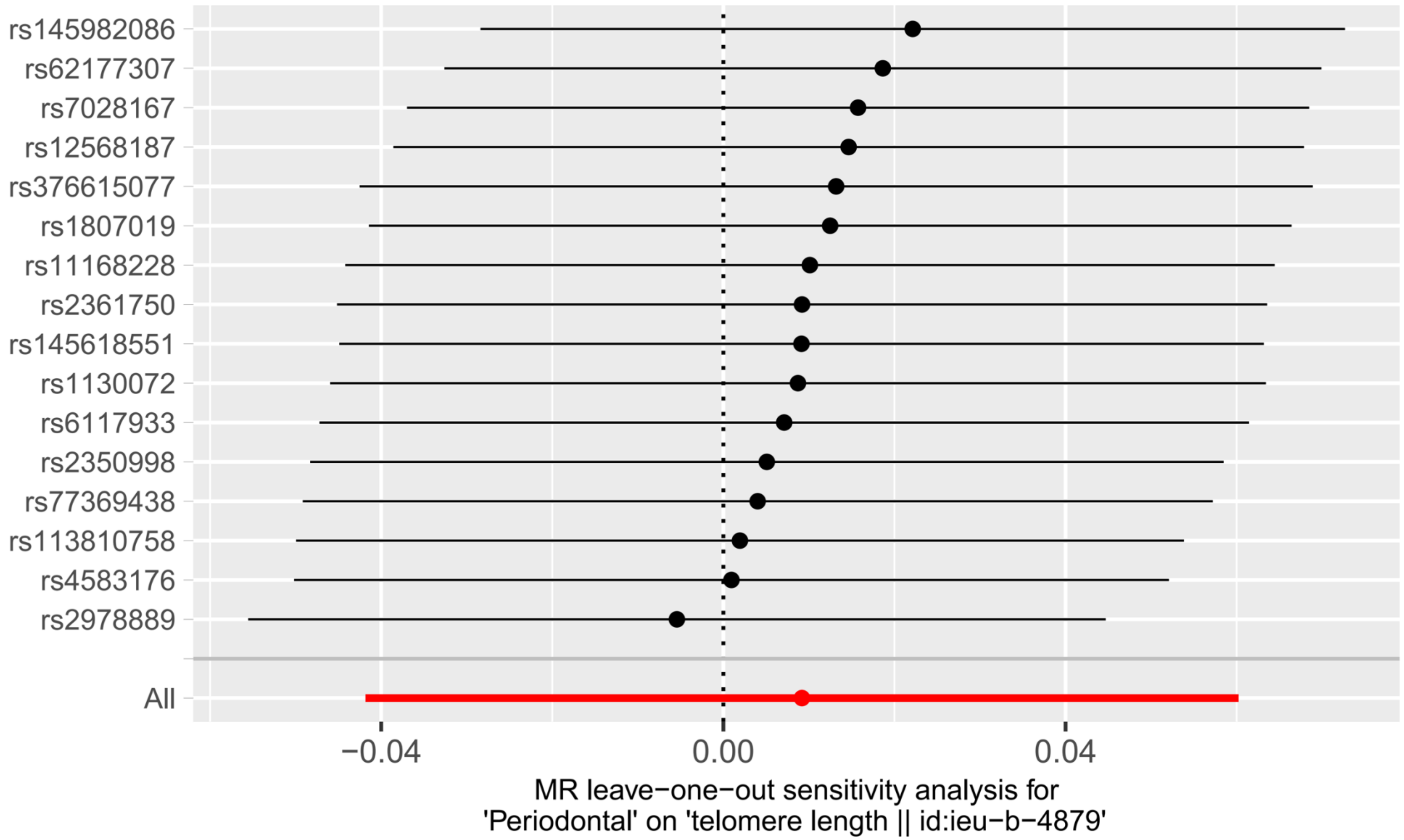

Supplementary figure 2, The forward MR: leave-one-out analyses.

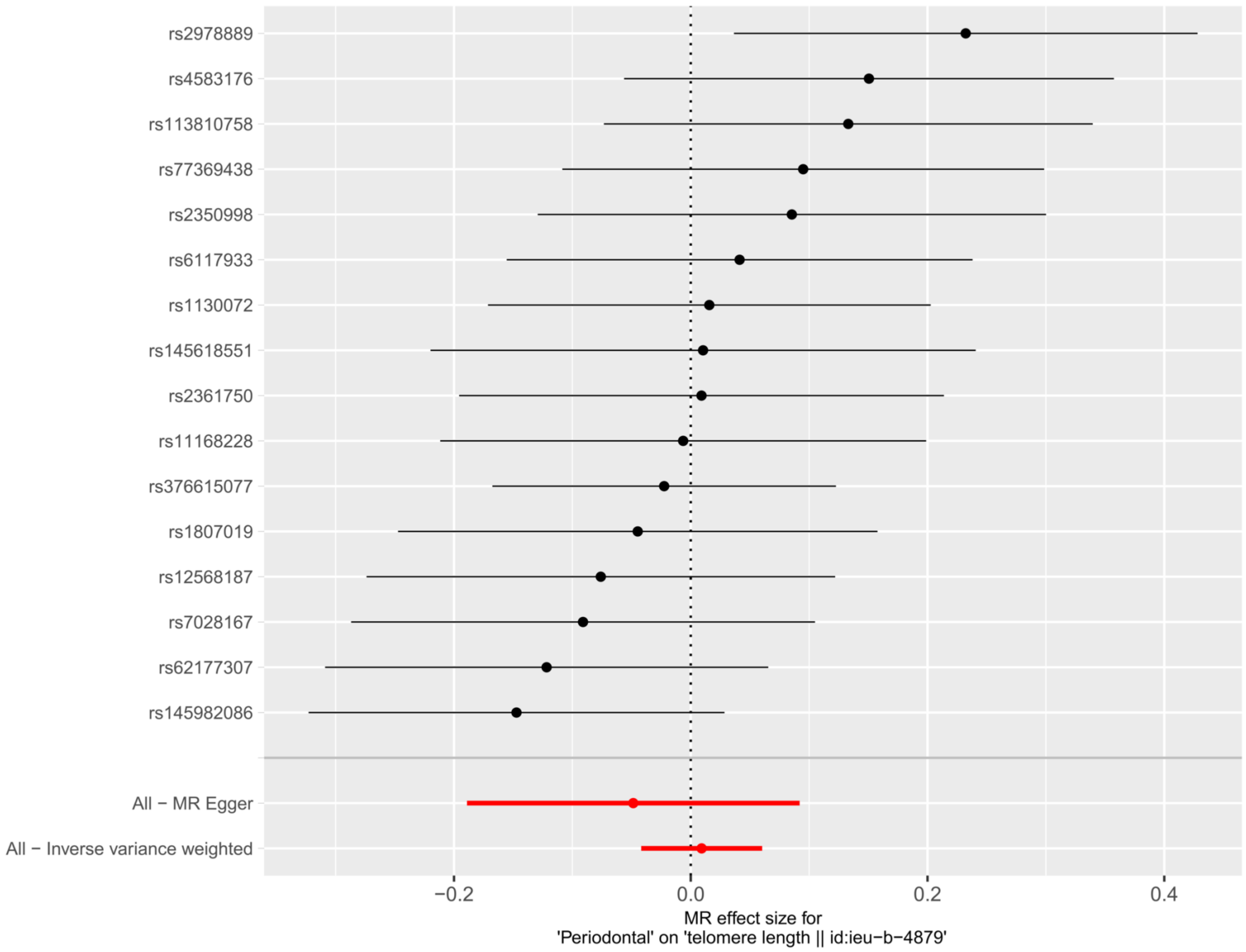

Supplementary figure 3, forward MR: the forest map, include the IVW and MR-Egger MR result.

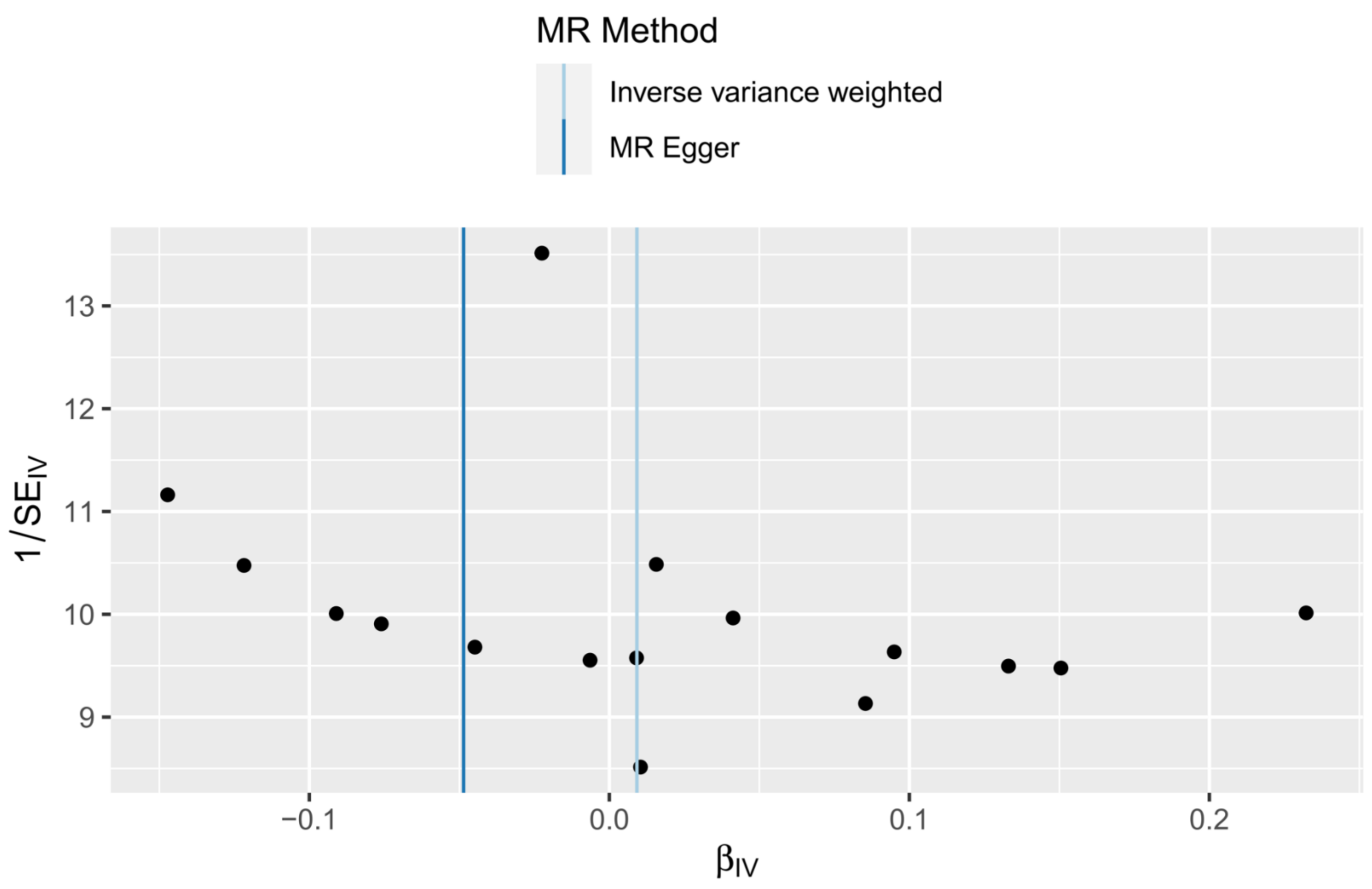

Supplementary figure 4, forward MR: A funnel plot.

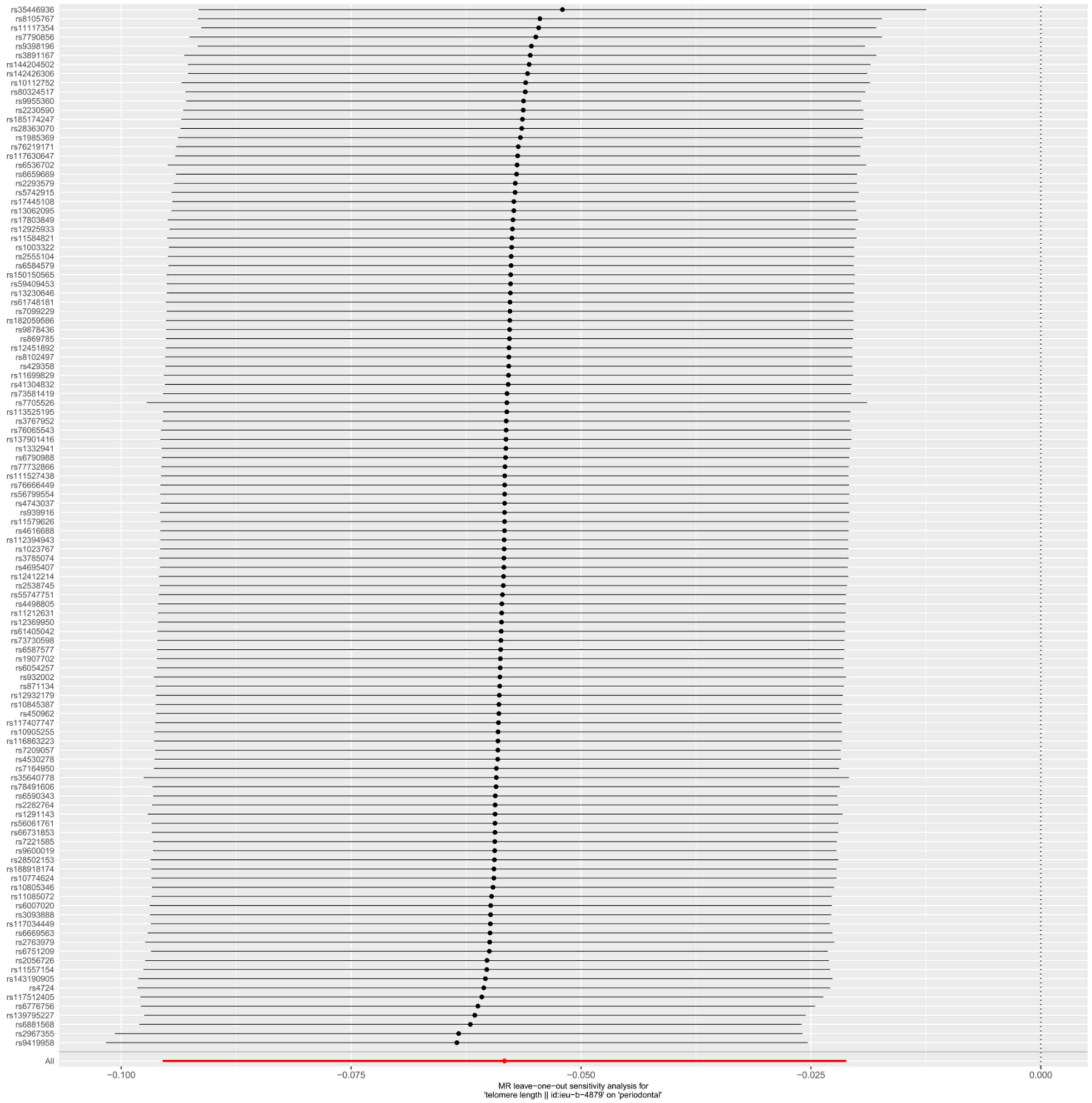

Supplementary figure 5, The reverse MR: Leave-one-out analyses.

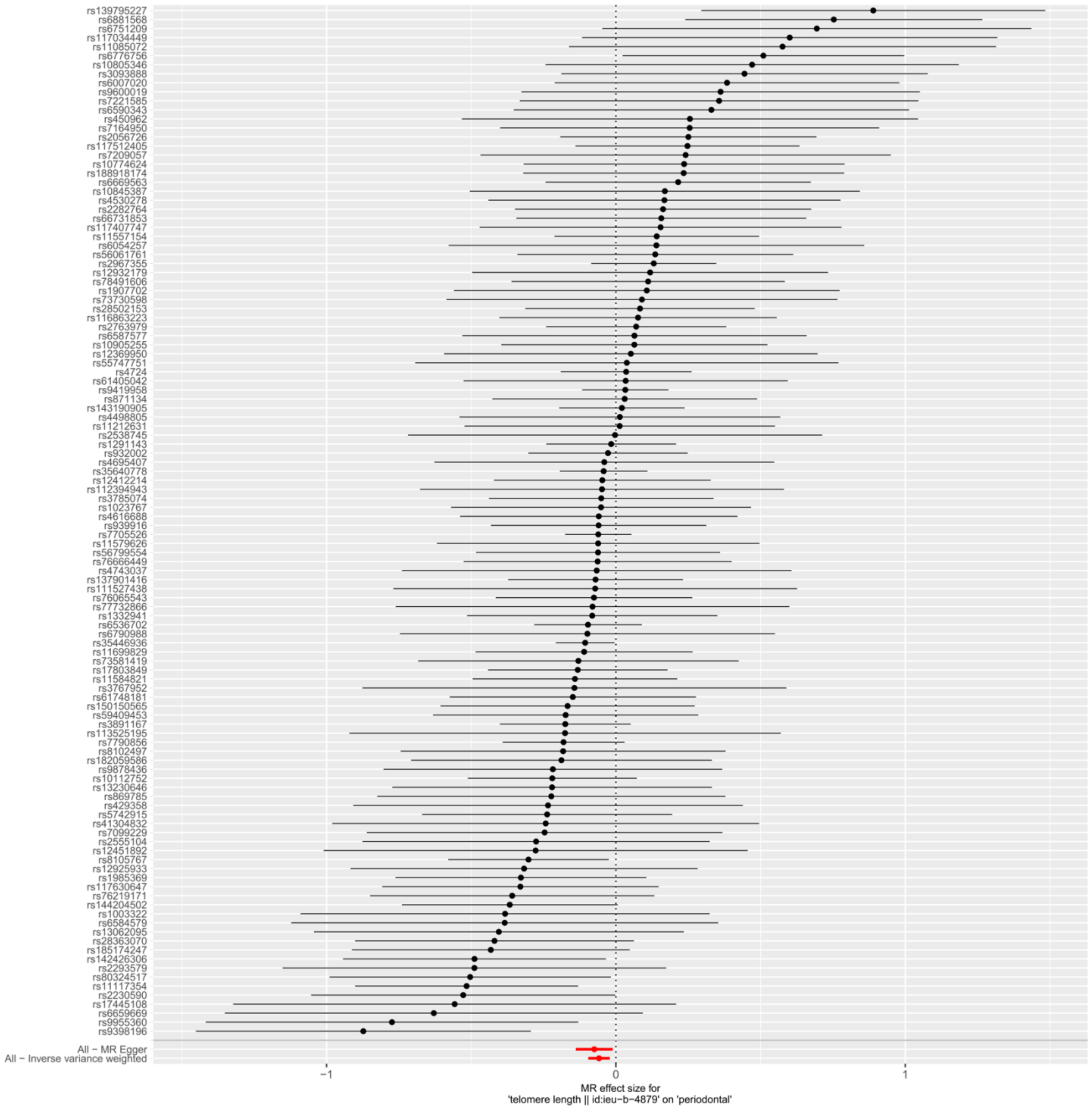

Supplementary figure 6, forward MR: the forest map, include the IVW and MR-Egger result.
